# Spatiotemporal evolution and drivers of carbon stock in arid oases: Multi-source assessment and scenario prediction in Wei-Ku oasis, China

**DOI:** 10.1016/j.isci.2026.115856

**Published:** 2026-04-24

**Authors:** Baisong An, Xuemei Wang, Changjiang Liu, Xinxue Feng

**Affiliations:** 1 College of Geographic Science and Tourism, Xinjiang Normal University, Urumqi 830017, China; 2 Linyi Vocational University of Science and Technology, Linyi 276000, China; 3 Xinjiang Laboratory of Lake Environment and Resources in Arid Zone, Urumqi 830017, China; 4 Weifang Private Economic Development Service Center, Weifang 261000, China

**Keywords:** environmental management, environmental monitoring, environmental science, global carbon cycle

## Abstract

Accurately assessing carbon stock (CS) in arid oasis ecosystems is vital for the global carbon cycle and regional “dual carbon” goals. This study integrates field surveys, satellite imagery, climate data, machine learning, In VEST model, and the optimal parameters-based geographical detector model to analyze the Wei-Ku Oasis. The results indicate that: (1) carbon density ranked as follows: garden land (27,00.62 gm^−2^) > forest-grassland (2,386.31 gm^−2^) > arable land (1,940.43 gm^−2^) > construction land (987.93 gm^−2^) > unused land (869.86 gm^−2^) > water bodies. (2) Total CS fluctuated from 2010 to 2022, with a net increase of 1.301 × 10^6^ t. By 2028, only the ecological protection scenario will increase CS. (3) Vegetation cover ($\bar{q}$ = 0.356) is the dominant driver of CS spatial heterogeneity, with interactions among factors enhancing explanatory power. This study provides an integrated assessment framework and scientific evidence for land use optimization and ecological strategy development in arid regions.

## Highlights

• Integrating field and climate data can improve the work of carbon stock estimation
• Ecological protection scenario has net carbon stock gain, informing sustainable planning
• Vegetation cover drives oasis carbon stock, synergizing with PET, elevation, and soil type

## Graphical abstract

**Figure:**
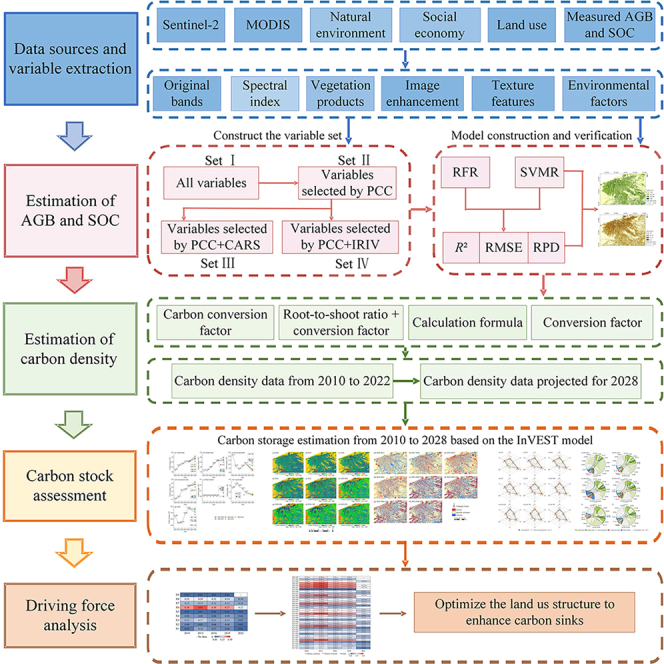

## Introduction

With the continuous advancement of urbanization and industrialization, land use (LU) patterns have undergone significant changes, resulting in a sharp increase in greenhouse gas emissions—particularly carbon dioxide. This trend not only exacerbates global warming but also triggers a series of environmental consequences, including more frequent extreme weather events and a dramatic decline in biodiversity.^1^^,^^2^^,^^3^ These issues pose serious threats to ecosystem stability and present growing challenges to the future of human society.^4^ In 2020, China announced its “dual carbon” strategic goals, committing to peaking carbon dioxide emissions by 2030 and achieving carbon neutrality by 2060. In this context, accurately assessing terrestrial carbon stock (CS) and enhancing carbon sink potential have become central topics in academic research.^5^ As a key driver of CS dynamics, LU directly influences the carbon source/sink balance of terrestrial ecosystems by altering surface cover. Therefore, estimating CS and analyzing its driving mechanisms are essential for accurately evaluating the carbon sink potential of terrestrial ecosystems, formulating effective carbon reduction policies, and improving climate models.^6^^,^^7^ Such efforts also contribute to a deeper understanding of the global carbon cycle, thereby supporting climate change mitigation and the pursuit of carbon neutrality.

Arid and semi-arid regions cover approximately one-third of the global land area. Within these regions, oases serve as critical ecological and economic zones characterized by unique hydrological and carbon cycling processes. In oasis areas, the interplay of water scarcity, salinization risk, and intensive human activities makes these ecosystems highly sensitive to LU changes, often weakening their carbon sink function and carbon fixation capacity. Previous studies have underscored the importance of understanding CS dynamics to support sustainable land management.^8^^,^^9^^,^^10^ Despite these efforts, research that integrates long-term, high-resolution field measurements, multi-source remote sensing data, and forward-looking scenario-based simulations to assess and project CS dynamics in arid oases remains scarce. Such integrated approaches are crucial for developing robust management strategies under future climate and LU uncertainties. Given the ecological fragility of arid oases and their pronounced sensitivity to climate change and anthropogenic disturbance, scenario-based projections are indispensable for guiding sustainable oasis management. Scenario analysis enables the evaluation of potential impacts from different development pathways on future CS dynamics, offering valuable insights for policymakers aiming to design LU strategies that enhance carbon sinks while balancing socioeconomic demands. Thus, the inclusion of multi-scenario prediction is a core component of this study, addressing the urgent need for proactive planning in vulnerable oasis ecosystems.

Currently, two main approaches are employed to estimate CS in terrestrial ecosystems: ground-based measurements and model simulations.^11^ Ground-based CS estimation primarily relies on field sampling and flux observations. Among these, field sampling—including soil organic carbon surveys and vegetation biomass assessments—serves as the foundational method.^12^ This approach yields the most accurate CS data to date and is often used to validate results from other estimation techniques. However, field sampling has notable limitations; it is time-consuming and labor-intensive, which constrains its applicability in large-scale CS assessments.^13^ Flux observations, typically conducted using eddy covariance systems, provide an effective tool for monitoring CS dynamics over long time periods. Nevertheless, this method is limited by the number of observation sites, reducing its representativeness and accuracy at regional scales.^14^ In contrast, model simulation methods offer greater flexibility for CS research across different spatial scales. Commonly used models include the Bookkeeping (BK) model,^15^ Biome-BGC,^16^^,^^17^ Denitrification-Decomposition (DNDC),^18^^,^^19^ and the Carnegie-Ames-Stanford Approach (CASA),^20^^,^^21^ all of which have been widely applied in CS studies. While these models produce relatively accurate CS estimates, their application is often constrained by model complexity and difficulties in parameter acquisition. In recent years, the InVEST (Integrated Valuation of Ecosystem Services and Trade-offs) model has gained popularity for quantitative CS assessment due to its high operational efficiency, low data requirements, and strong spatial visualization capabilities.^22^^,^^23^ For instance, Huang et al.^24^ used the model to assess CS dynamics in China’s Turpan-Hami Basin under various scenarios; Lin et al.^25^ applied it to evaluate CS in the Ningxia section of the Yellow River Basin; and Bacani et al.^26^ and Zhu et al.^27^ employed the InVEST model in forest CS studies. Both of the latter studies indicated that forest protection significantly enhances the carbon sequestration capacity of terrestrial ecosystems. Scientific and rational territorial spatial planning is thus critical for enhancing carbon sink potential, and the InVEST model has been widely validated as an effective and reliable tool for CS research.

For CS assessments, the InVEST model primarily relies on LU and carbon density (CD) data. LU data are typically derived from publicly accessible remote sensing products (e.g., China Land Cover Dataset [CLCD],^28^ global land-cover product [GLC]_ with fine classification system at 30 m [FCS30]^29^), while CD data are mainly obtained from the literature. These data sources, however, can constrain the accuracy of CS estimates in specific regions. More accurate and applicable LU data can be generated using classification systems developed from regional field surveys. Furthermore, when CD data are unavailable, researchers often rely on values from geographically similar regions—a practice that may introduce errors due to inherent regional differences.^30^^,^^31^ Therefore, integrating remote sensing data with ground-based sampling can enhance the accuracy of regional CD estimation and support high-precision CS mapping. In summary, obtaining high-precision LU and CD data through field surveys can significantly improve the accuracy of the InVEST model in regional CS assessments, thereby providing a solid foundation for research on the terrestrial carbon cycle.

Terrestrial CS is shaped by the combined effects of natural and anthropogenic factors. Natural factors include topography, climate, and vegetation cover (VC), while human factors encompass LU change, gross domestic product, and other socioeconomic indicators. Due to the interplay of multiple drivers, CS in terrestrial ecosystems exhibits considerable spatial heterogeneity and dynamic spatiotemporal patterns. Among these factors, LU plays a particularly critical role by directly altering surface cover and thereby influencing the temporal and spatial distribution of CS. Current research on the driving mechanisms of CS mainly focuses on the relationship between LU and CS.^32^^,^^33^ For example, Wang et al.^34^ and An et al.^35^ used the Patch-generating Land Use Simulation (PLUS) model and the InVEST model to project future CS trends and underlying drivers in China’s Changchun-Jilin-Tumen region and the Yangtze River basin, respectively. Given the complexity of factors influencing CS changes, clarifying their respective roles is essential for a deeper understanding of carbon sink potential. This complexity is especially pronounced in arid and semi-arid regions, which account for one-third of the world’s land area.^36^ In these regions, CS not only exhibits significant spatial variability but is also governed by intricate driving mechanisms.^37^^,^^38^ Therefore, investigating CS changes in arid oases and their underlying drivers offers valuable insights into the global carbon cycle.^39^ To better elucidate the mechanisms governing carbon dynamics in fragile arid ecosystems, this study focuses on a typical arid oasis in China—the Weigan-Kuqa River Delta oasis (hereafter referred to as the “Wei-Ku Oasis”). This region has experienced significant LU changes, primarily driven by agricultural expansion and urbanization. Its ecological vulnerability and water resource constraints make it an ideal case for exploring CS dynamics under different development pathways. By integrating multi-temporal field measurement data, remote sensing imagery, historical and projected climate data, and applying machine learning and the optimal parameters-based geographical detector (OPGD) model, this study aims to accurately quantify CS dynamics in the Wei-Ku Oasis from 2010 to 2028 and reveal their driving mechanisms.

This study establishes three core research objectives: (1) to precisely characterize the spatiotemporal evolution of CS in the oasis ecosystem; (2) to simulate and assess future CS dynamics under different scenarios; and (3) to elucidate the driving mechanisms underlying the spatial differentiation of CS and identify dominant factors and their interactions. By integrating historical assessments with multi-scenario predictions, this research provides a replicable framework for comprehensive carbon assessment in oasis ecosystems. The findings are expected to offer scientific guidance for optimizing LU structures, informing ecological protection strategies, and supporting the implementation of China’s “dual carbon” goals in arid regions.

## Results

### CD estimation

#### AGB estimation

**Figure 1:**
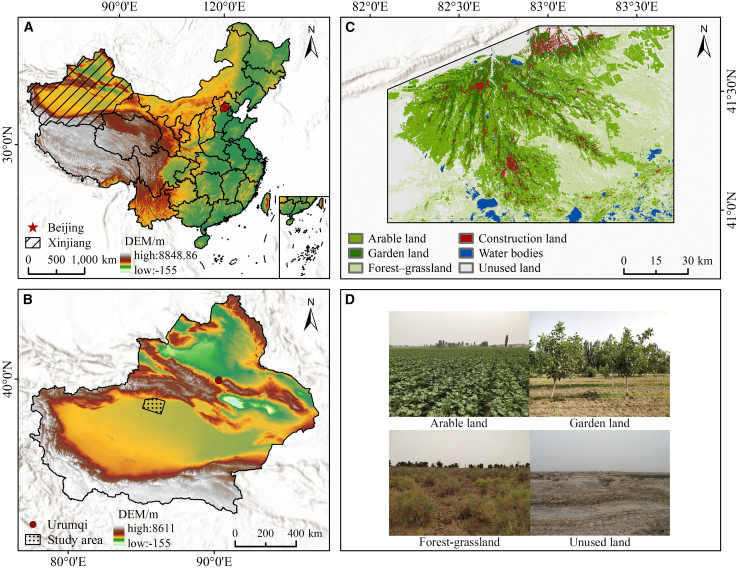
Wei-Ku Oasis (A) China; (B) Xinjiang; (C) study area; and (D) investigate photos

The study area is the Wei-Ku Oasis (Figure 1). The measured data and remote sensing imagery from the study area in 2019 were utilized to estimate and invert aboveground biomass (AGB) for CD assessment. A total of 162 biomass samples were allocated into a training set and a validation set at a ratio of 7:3 utilizing the Sample set Partitioning based on joint X-Y distances (SPXY) algorithm, followed by statistical analysis of the samples. Table 1 illustrates that the AGB of the 162 samples varied from 5.8 gm^−2^ to 3,416.5 gm^−2^, with a mean of 4,37.7 gm^−2^ and a standard deviation of 631.3 gm^−2^. The analysis indicates a significant disparity in AGB values, characterized by a prevalence of low-value data points.

**Table 1:** AGB statistics

Sample type	Sample size	AGB/(g·m^−2^)			
		Maximum	Minimum	Average	Standard deviation
Total sample	162	3,416.52	5.83	437.74	631.27
Training set	113	3,416.52	5.83	527.41	669.14
Validation set	49	3,299.98	5.85	230.96	478.88

**Figure 2:**
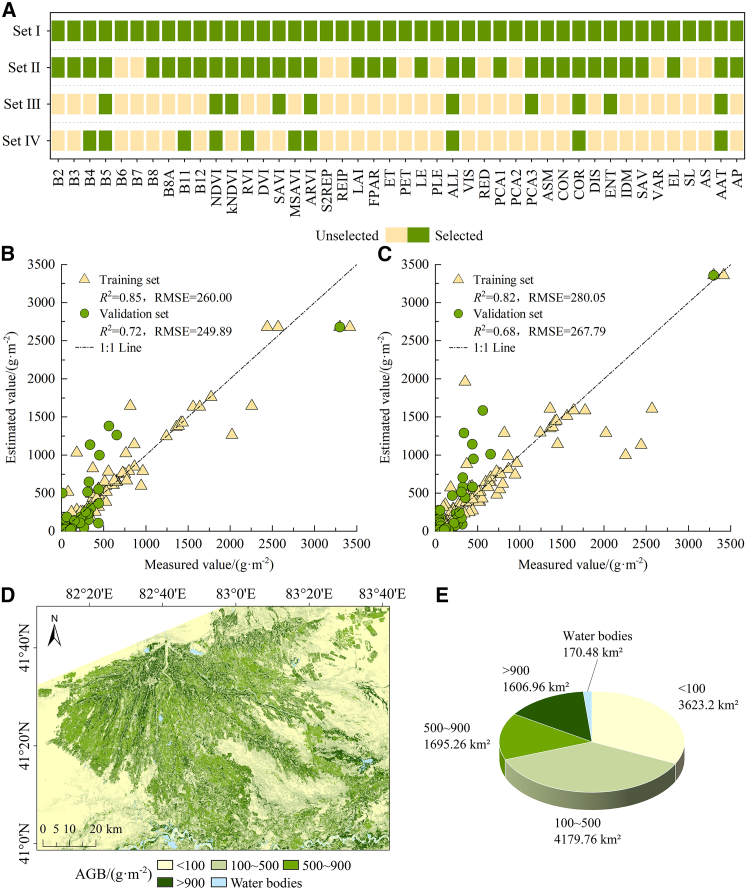
AGB estimation (A) Combination of variables used. (B) Scatterplot of set IV-RFR model. (C) Scatterplot of set IV-SVMR model. (D) AGB inversion map in 2019. (E) Area of each category. Set Ⅰ is all variables; set Ⅱ is the variables selected by PCC; set Ⅲ is the variables selected by PCC+CARS; and set Ⅳ is the variables selected by PCC+IRIV.

Four variable combinations for AGB estimation were identified through the integration of various variable selection methods. Figure 2A illustrates that Set I, Set II, Set III, and Set IV comprise 44, 33, 10, and 10 variables, respectively. B5, normalized difference vegetation index (NDVI), atmospherically resistant vegetation index (ARVI), sum of B2–B8, B8A, B11, B12 (ALL), correlation (COR), and annual average temperature (AAT) were retained in four combinations, while B4, B11, Kernel normalized difference vegetation index (kNDVI), ratio vegetation index (RVI), soil-adjusted vegetation index (SAVI), modified soil-adjusted vegetation index (MSAVI), the first three principal components from principal component analysis (PCA3) and entropy (ENT) were retained in three combinations.

Four groups of AGB-related variables were combined to construct AGB estimation models using random forest regression (RFR) and support vector machine regression (SVMR). Table 2 presents the modeling accuracy. In comparing the overall estimation effects of the two models, the RFR model demonstrates superior performance relative to the SVMR model. Analysis of variable combinations indicates that the exactness of the AGB estimation model developed using competitive adaptive reweighted sampling (CARS) and iterative retained information variable (IRIV) secondary selection variables surpasses that of the basic Pearson’s correlation coefficient (PCC) selection and the no selection scenario. Notably, the PCC+IRIV selection yields the highest estimation accuracy. The RFR model, developed through the integration of PCC and IRIV selection variables, demonstrates optimal performance. In comparison to the SVMR model developed using PCC+IRIV selected variables, the validation set exhibited an increase in *R*^*2*^ by 0.04, a decrease in root-mean-square error (RMSE) by 17.90 gm^−2^, and an increase in relative percent deviation (RPD) by 0.13. Scatterplots were created to examine the exactness of AGB estimation, with measured values on the horizontal axis and estimated values on the vertical axis, facilitating a comparison of optimal estimation results between the two models. Figures 2B and 2C illustrate that the sample points of the Set Ⅳ-RFR model are positioned nearer to the 1:1 line compared to those of the Set Ⅳ-SVMR. Analysis of Table 2 and Figures 2B and 2C indicates that the Set IV-RFR model is the most effective estimation model for AGB.

**Table 2:** Comparison of AGB estimation accuracy

Model	Variable combination	Training set			Validation set		
		*R*^2^	RMSE/(g·m^−2^)	RPD	*R*^2^	RMSE/(g·m^−2^)	RPD
RFR	set Ⅰ	0.77	320.70	2.09	0.57	311.76	1.54
	set Ⅱ	0.77	317.60	2.11	0.60	299.73	1.60
	set Ⅲ	0.81	292.30	2.29	0.71	255.38	1.88
	set Ⅳ	0.85	260.00	2.57	0.72	249.89	1.92
SVMR	set Ⅰ	0.75	331.00	2.02	0.52	327.29	1.46
	set Ⅱ	0.74	338.97	1.97	0.58	307.00	1.56
	set Ⅲ	0.79	305.87	2.19	0.60	299.74	1.60
	set Ⅳ	0.82	280.05	2.39	0.68	267.79	1.79

Set Ⅰ is all variables; set Ⅱ is the variables selected by PCC; set Ⅲ is the variables selected by PCC+CARS; and set Ⅳ is the variables selected by PCC+IRIV.

Based on the estimation results, the AGB spatial inversion was performed using the R language, as indicated by the estimation results of the Set Ⅳ-RFR model (Figures 2D and 2E). In 2019, the AGB inversion value within the study area varied between 6.2 gm^−2^ and 2,679.8 gm^−2^. AGB exhibits notable spatial differentiation characteristics. The AGB within the oasis is markedly greater than that found outside, exhibiting a declining trend from the interior of the oasis to the surrounding desert. The AGB is elevated in the southern region. The Tarim River, situated in the south, possesses abundant water resources that facilitate vegetation growth. The analysis of LU distribution indicates that garden land exhibits the highest AGB, followed by arable land, forest-grassland, and finally, unused land, which has the lowest AGB.

#### SOC estimation

Data from the study area in 2019 were utilized to estimate soil organic carbon (SOC), leading to the creation of an SOC inversion map for assessing soil CD. In total, 96 soil samples were allocated into a training set comprising 70% and a validation set consisting of 30% through the application of the SPXY algorithm. Subsequently, both the complete sample set and the partitioned data underwent statistical analysis (Table 3). The SOC content in the study area ranged from 0.67 to 10.20 gkg^−1^. The average values for the total sample, training set, and validation set were 4.99, 4.87, and 5.26 gkg^−1^, respectively, suggesting a relative deficiency in soil nutrients.

**Table 3:** SOC statistics

Sample type	Sample size	SOC/(g·kg^−1^)			
		Maximum	Minimum	Average	Standard deviation
Total sample	96	10.20	0.67	4.99	2.10
Training set	67	10.20	0.67	4.87	2.23
Validation set	29	8.21	1.39	5.26	1.78

**Figure 3:**
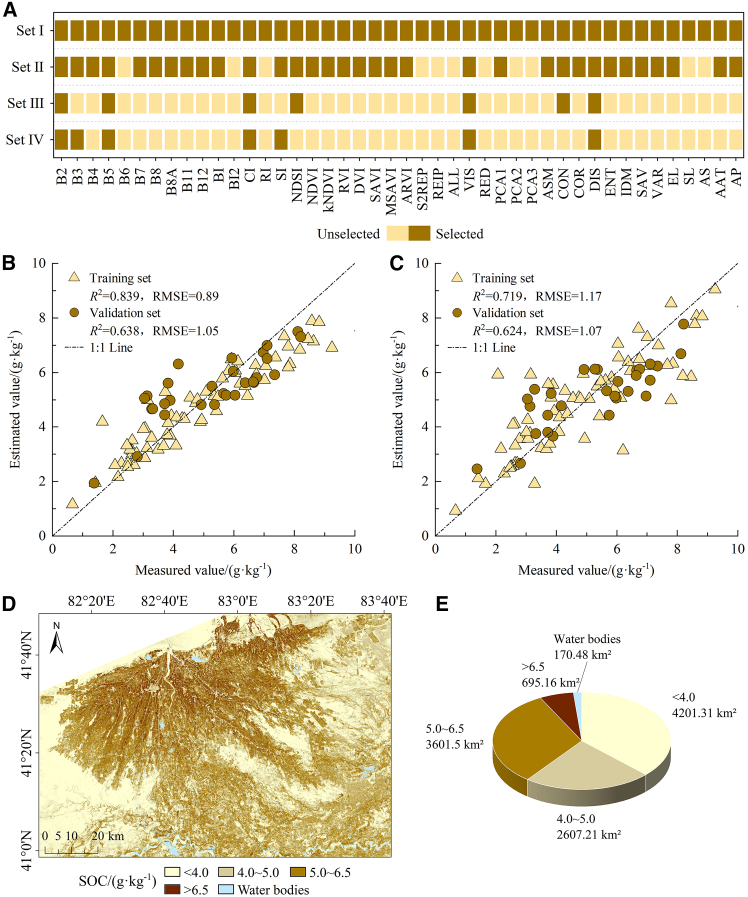
SOC estimation (A) Combination of variables used. (B) Scatterplot of set Ⅱ-RFR model. (C) Scatterplot of set Ⅱ-SVMR model. (D) SOC inversion map in 2019. (E) Area of each category. Set Ⅰ is all variables; set Ⅱ is the variables selected by PCC; set Ⅲ is the variables selected by PCC+CARS; and set Ⅳ is the variables selected by PCC+IRIV.

Figure 3A presents four combinations of variables for the estimation of SOC. Set I contains 44 variables, Set II contains 33 variables, Set III contains 7 variables, and Set IV contains 7 variables. B3, SI, Normalized Difference Salinity Index (NDSI), and contrast (CON) are retained on three occasions. Nevertheless, B2, B5, CI, VIS (B2 + B3 + B4), and dissimilarity (DIS) are collectively retained by four combinations, suggesting a significant relationship between these five variables and SOC.

Based on the RFR and SVMR models, the SOC estimation model was constructed with four groups of SOC-related variables as independent variables and SOC as the dependent variable. Table 4 presents the modeling accuracy. The model that incorporates PCC selection variables demonstrates a higher accuracy compared to the AGB estimation results, while the secondary selection using CARS and IRIV algorithms does not enhance the performance of the SOC estimation model. The RFR model demonstrates superior performance compared to the SVMR model in the context of SOC estimation. The analysis indicates that the SOC estimation model, which integrates PCC-selected variables with RFR, achieves the highest accuracy. The training set and validation set exhibit *R*^*2*^ values of 0.84 and 0.64, respectively, along with RMSE values of 0.89 and 1.05 gkg^−1^, and RPD values of 2.51 and 1.69, respectively. To analyze the accuracy of SOC estimation, the optimal estimation results from the two models were selected for scatterplot representation. Figures 3B and 3C illustrate that the sample points of the Set II-RFR model are significantly closer to the 1:1 line compared to those of the Set II-SVMR, suggesting superior estimation capability of the former model. Analysis of Table 4 and Figures 3B and 3C indicates that the Set II-RFR model is the most effective estimation model for SOC.

**Table 4:** Comparison of SOC estimation accuracy

Model	Variable combination	Training set			Validation set		
		*R*^2^	RMSE/(g·kg^−1^)	RPD	*R*^2^	RMSE/(g·kg^−1^)	RPD
RFR	set Ⅰ	0.76	1.08	2.06	0.63	1.07	1.67
	set Ⅱ	0.84	0.89	2.51	0.64	1.05	1.69
	set Ⅲ	0.68	1.26	1.77	0.55	1.17	1.51
	set Ⅳ	0.76	1.08	2.07	0.53	1.20	1.48
SVMR	set Ⅰ	0.74	1.13	1.97	0.61	1.10	1.62
	set Ⅱ	0.72	1.17	1.90	0.62	1.07	1.66
	set Ⅲ	0.70	1.22	1.83	0.46	1.28	1.39
	set Ⅳ	0.73	1.15	1.95	0.47	1.27	1.40

Set Ⅰ is all variables; set Ⅱ is the variables selected by PCC; set Ⅲ is the variables selected by PCC+CARS; and set Ⅳ is the variables selected by PCC+IRIV.

Based on the Set Ⅱ-RFR model, the SOC in 2019 was spatially inverted (Figures 3D and 3E). The SOC inversion values in the study area varied between 1.08 and 8.94 gkg^−1^. The SOC content was elevated in areas with significant vegetation coverage, including oases, riverbanks, lakes, and forest-grassland interfaces, indicating the critical role of vegetation in sustaining soil nutrients. The analysis of LU distribution reveals that the SOC content decreases in the following order: garden land, arable land, forest-grassland, and unused land.

#### Spatial distribution and multi-temporal parameters of CD

**Figure 4:**
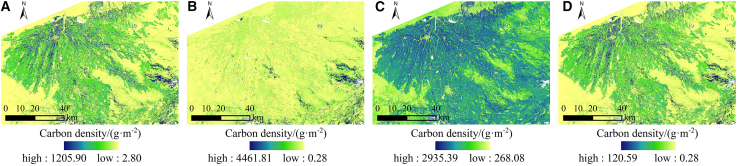
CD map in 2019 (A) Aboveground CD; (B) underground CD; (C) soil CD; and (D) dead organic CD.

Combining a variety of methods, including the carbon conversion coefficient, root-to-shoot ratio, soil CD calculation formula, and green coverage rate, the CD in 2019 was estimated. Figure 4 illustrates that the CD values for aboveground, underground, soil, and dead organic matter in the central region of the oasis exceed those found in the peripheral areas. The soil CD in the oasis periphery is higher than that of aboveground, underground, and dead organic matter.

**Figure 5:**
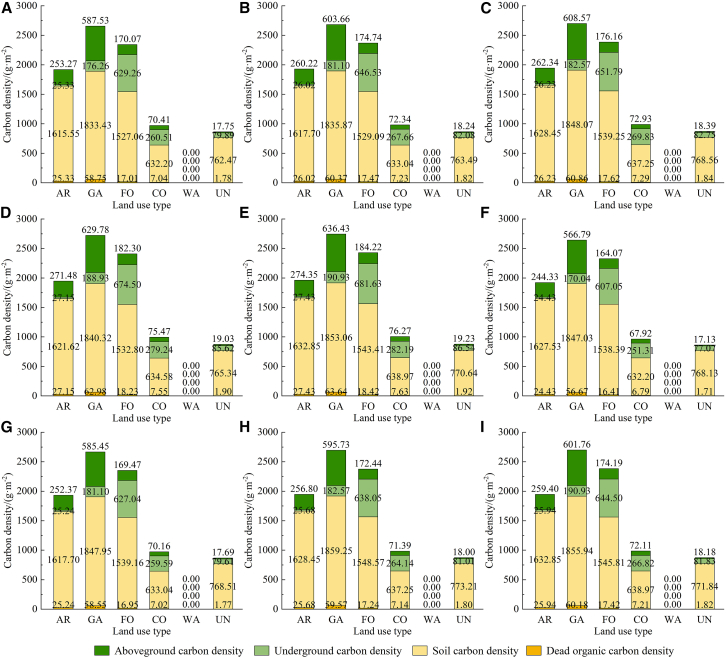
CD of each LU type from 2010 to 2028 (A) 2010; (B) 2013; (C) 2016; (D) 2019; (E) 2022; (F) 2028NDS; (G) 2028EDS; (H) 2028CDS; and (I) 2028EPS). AR, arable land; GA, garden land; FO, forest-grassland; CO, construction land; WA, water bodies; UN, unused land.

Figure 4 was used to calculate the average CD of each LU type in 2019, thereby generating the CD parameters for 2019 necessary for the InVEST model. Historical and projected temperature and precipitation data were utilized to adjust the CD from 2019 to obtain the CD in 2010, 2013, 2016, 2022, and 2028. Figure 5 illustrates the multi-period CD parameters for the years 2010–2028. Analysis of data across multiple periods indicates that the aboveground CD, soil CD, and dead organic CD in garden land surpass those of other LU types. The underground CD in forest-grassland ecosystems exceeds that of other LU types. The total CD of each LU type is ranked as follows: garden land, forest-grassland, arable land, construction land, unused land, and water bodies.

### CS assessment

#### Temporal change analysis

**Figure 6:**
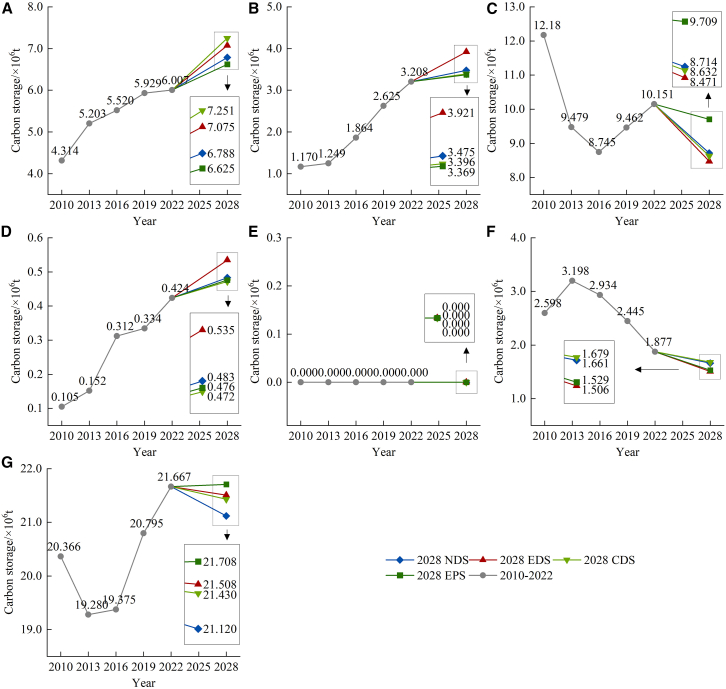
Temporal change trend of CS from 2010 to 2028 (A) Arable land; (B) garden land; (C) forest-grassland; (D) construction land; (E) water bodies; (F) unused land; and (G) total.

Combined with multi-period LU and CD data, the InVEST model was used to estimate and simulate CS from 2010 to 2028. Figure 6 presents the statistical results. The total CS exhibited a decline from 2010 to 2013, followed by an increase from 2013 to 2022, culminating in a net increase of 1.301 × 10^6^ t. The CS of arable land, garden land, and construction land exhibited a continuous increase from 2010 to 2022, with increments of 1.693 × 10^6^, 2.038 × 10^6^, and 0.319 × 10^6^ t, respectively. The CS of forest-grassland initially declined and subsequently rose, resulting in a net decrease of 2.029 × 10^6^ t. The CS of unused land initially increased, followed by a decrease, resulting in an overall reduction of 0.721 × 10^6^ t. The change in CS in garden land was the most striking within all LU types. In the projected future scenario, the total CS of natural development (NDS), economic development (EDS), and cropland development (CDS) is anticipated to decline relative to 2022, whereas the total CS of ecological protection scenario (EPS) is expected to rise, reflecting an overall increase of 0.041 × 10^6^ t.

#### Spatial change analysis

**Figure 7:**
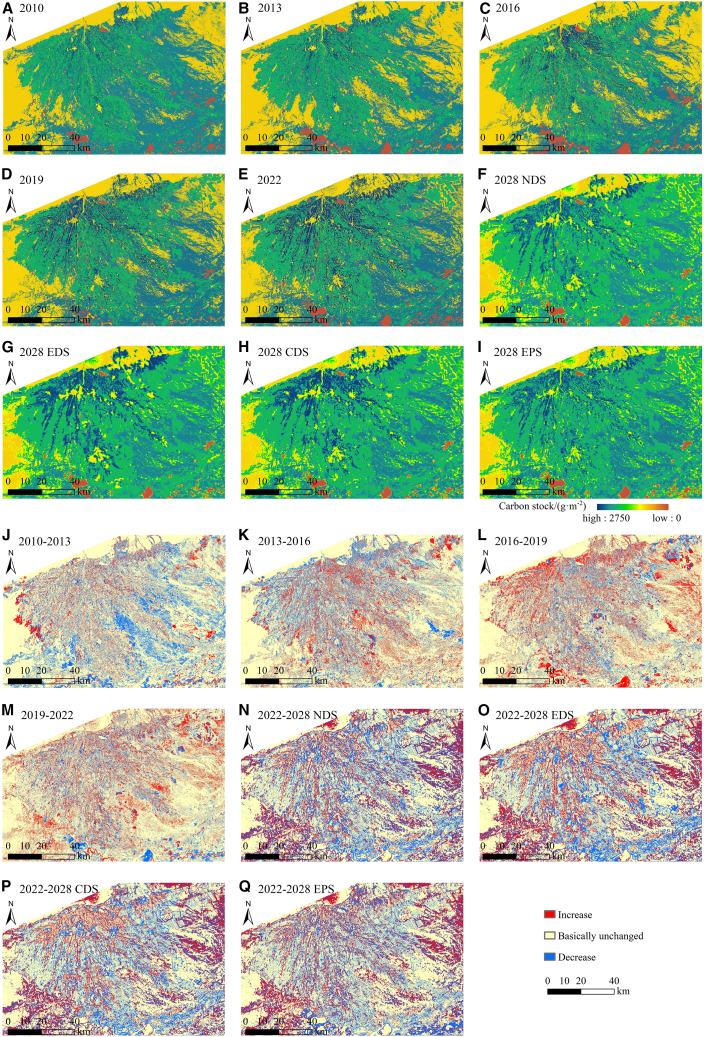
Distribution pattern and spatial change trend of CS from 2010 to 2028 (A–I) Distribution pattern maps. (J–Q) Change trend maps.

The spatial distribution pattern of CS is shown in Figures 7A–7I. From 2010 to 2028, the area exhibited a spatial distribution pattern characterized by high CS within the oasis interior and the oasis-desert transition zone, while demonstrating low CS in the desert region beyond the oasis. The CS in the lower section of the alluvial fan was relatively low in 2013, 2016, and 2019. The high-value CS area in the upper section of the oasis exhibited an expansion trend.

To elucidate the spatial variation characteristics of CS, the CS data from the two periods were differenced and reclassified into three categories: decreasing, essentially unchanged (change amount within ±5%), and increasing (Figures 7J–7Q). Between 2010 and 2013, there was an increase in CS in the western region of the oasis, while a significant decrease in CS occurred in the lower section of the alluvial fan. This change was associated with the expansion of arable land and the reduction of forest-grassland, respectively. Between 2013 and 2016, the garden land in the upper section of the oasis expanded, the arable land in the northeast corner was developed, and the forest-grassland in the lower section of the alluvial fan recovered, resulting in an increase in CS across these three areas. Between 2016 and 2022, the expansion of garden land in the upper section of the oasis and arable land in the northeast corner resulted in an increase in CS in that area. The largest area with unchanged CS occurred between 2010 and 2013, measuring 8,233.29 km^2^. The greatest area with increased CS was recorded from 2016 to 2019, totaling 1,985.11 km^2^. Conversely, the smallest area with decreased CS was observed from 2019 to 2022, amounting to 1,216.67 km^2^. Future predictions indicate that, according to the NDS and EDS from 2022 to 2028, numerous areas within the oasis have experienced a significant decrease in CS. The enlargement of urban and rural localities has triggered a diminution in CS. Between 2022 and 2028, the EDS and CDS indicate a decline in CS in the downstream areas of the oasis and near the Tarim River, attributed to the alteration of forest-grassland to arable land. From 2022 to 2028, the EPS indicates that forest-grassland areas are protected, resulting in no significant reduction in CS.

#### CS analysis based on different LU types

**Figure 8:**
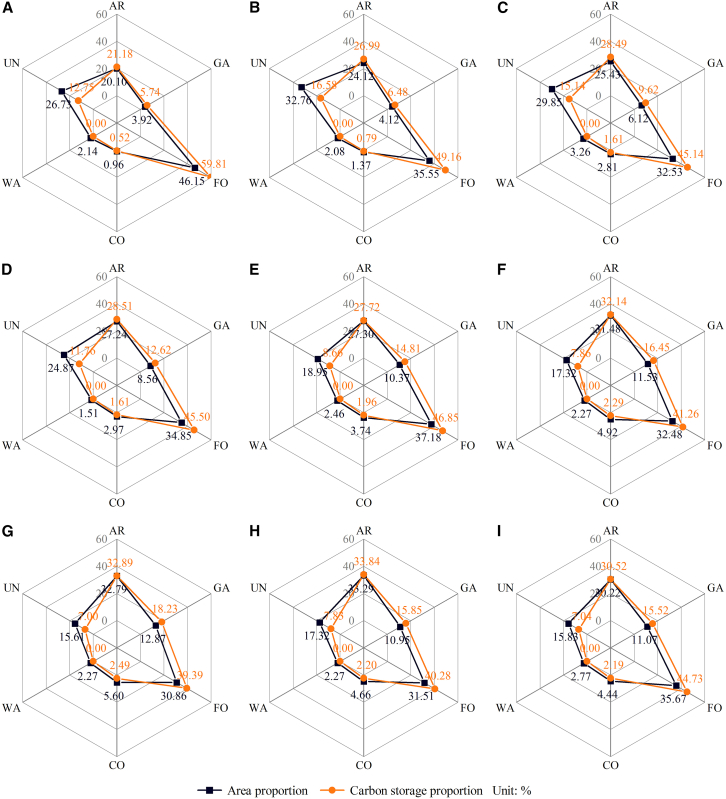
Area proportion and CS proportion of each LU type from 2010 to 2028 (A) 2010; (B) 2013; (C) 2016; (D) 2019; (E) 2022; (F) 2028 NDS; (G) 2028 EDS; (H) 2028 CDS; and (I) 2028 EPS. AR, arable land; GA, garden land; FO, forest-grassland; CO, construction land; WA, water bodies; UN, unused land.

This study compares the area ratio and CS ratio of each LU type from 2010 to 2028 (Figure 8) to measure the carbon fixation capacity of these LU types. The CS ratio of arable land from 2010 to 2022 exceeded the area ratio, suggesting a relatively high carbon fixation capacity of arable land. Between 2010 and 2022, the CS ratio of garden land was approximately 1.5 times greater than the area ratio, suggesting that garden land possesses a superior carbon fixation capacity compared to arable land. Compared with garden land and arable land, the carbon fixation capacity of forest-grassland was found to be lower than that of garden land but higher than that of arable land. The CS ratio of construction land, water bodies, and unused land in the five-period data were lower than the area ratio, suggesting a limited carbon fixation capacity for these three LU types. The carbon fixation capacity of each LU type in 2028 aligned with the data from 2010 to 2022.

**Figure 9:**
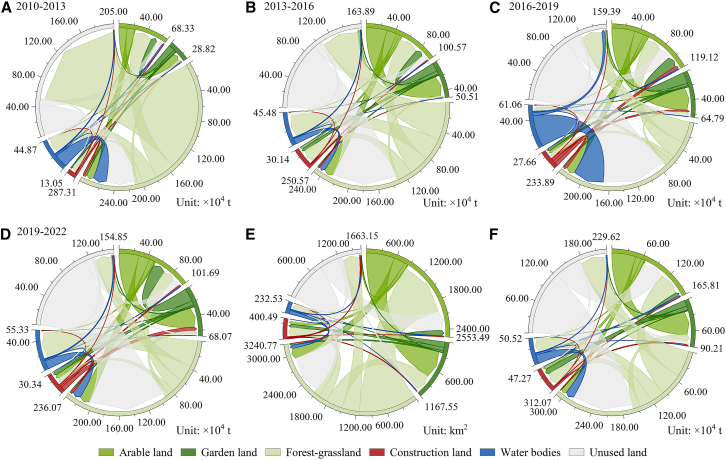
Changes in LU and CS from 2010 to 2022 (A–D) Variations in CS resulting from various LU conversions across distinct periods. (E) Alteration between different LU types from 2010 to 2022. (F) Changes in CS stemming from conversion of different LU types from 2010 to 2022.

Figures 9A–9D illustrate the variations in CS resulting from various LU conversions across distinct periods. Between 2010 and 2013, there was a significant reduction in CS attributed to the conversion of forest-grassland into unused land. Between 2013 and 2016, 2016 and 2019, and 2019 and 2022, CS experienced an increase primarily owing to the alteration of unused land into forest-grassland. Figures 9E and 9F illustrate that the rise in CS resulting from various LU conversions between 2010 and 2022 was predominantly associated with the alteration of unused land to forest-grassland and arable land. Specifically, 787.20 km^2^ of unused land was transformed into forest-grassland, contributing 1.233 × 10^6^ t of carbon, while 404.34 km^2^ was converted to arable land, contributing 0.445 × 10^6^ t of carbon. Simultaneously, a portion of the arable land was transformed into garden land, which possesses a higher carbon fixation capacity, resulting in an increase in CS of 0.467 × 10^6^ t. Between 2010 and 2022, various LU conversions resulted in an increase of 2.706 × 10^6^ t in CS, a decrease of 1.771 × 10^6^ t in CS, and a net increase of 0.935 × 10^6^ t in CS. The CD varies across periods, resulting in changes to the CS in regions with stable LU during each interval. In contrast to the alterations in CS resulting from various LU conversions, the modifications in CS within areas of stable LU exerted a lesser influence on the overall changes in total CS.

In addition to LU conversion, the changing trends in CS in forest-grassland and garden land are also influenced by oasis-specific agricultural practices. The initial decline in forest-grassland CS may be linked to water competition from expanding irrigated agriculture and historical overgrazing. Conversely, the substantial rise in garden land CS is not solely due to area expansion but also likely results from intensified management, including optimized irrigation, fertilization, and the cultivation of high-value perennial crops, which maintain high biomass and soil organic inputs.

### Analysis of driving forces of spatiotemporal heterogeneity of CS

**Figure 10:**
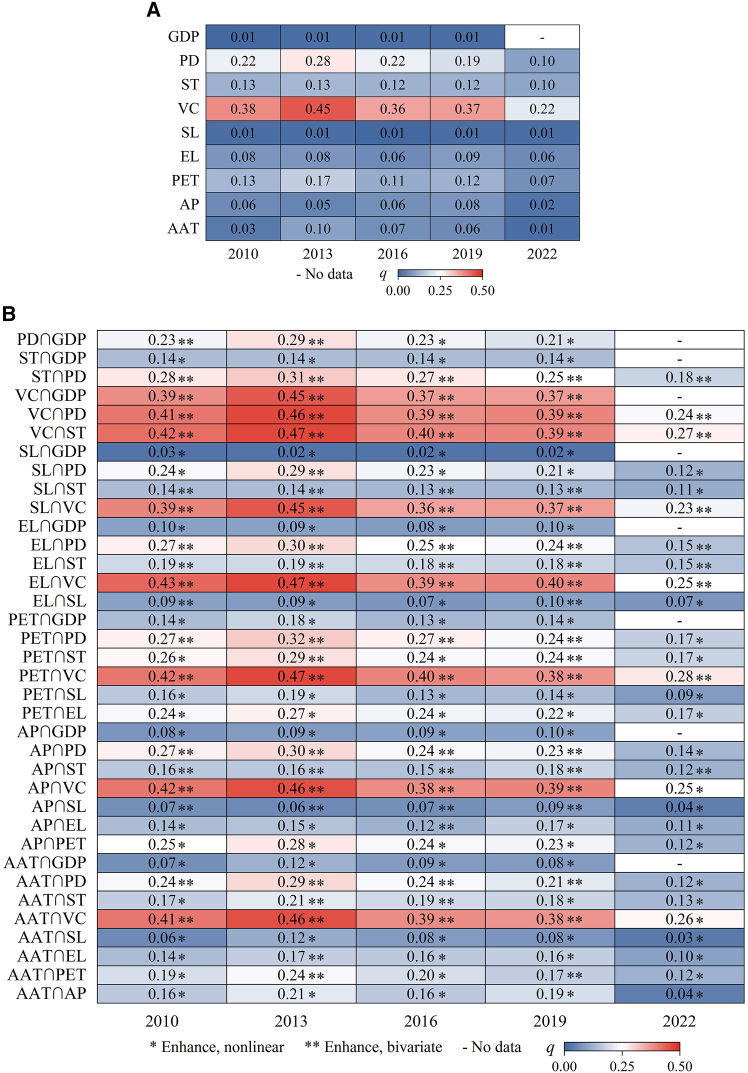
Driving force detection results (A) Factor detection results (*p* < 0.01). (B) Interactive detection results (*p* < 0.01). AAT, annual average temperature; AP, annual precipitation; PET, potential evapotranspiration; EL, elevation; SL, slope; VC, vegetation cover; ST, soil type; PD, population density; GDP, gross domestic product density.

The explanatory power (*q*) of factors driving CS is categorized as follows: *q* ≥ 0.3 indicates a main influence, 0.2 ≤ *q* < 0.3 signifies a strong influence, 0.1 ≤ *q* < 0.2 represents a medium influence, and *q* < 0.1 denotes a weak influence. Factor detection (Figure 10A) indicates that vegetation cover (VC) and population density (PD) consistently rank as the primary and secondary contributors to explanatory power each year. VC significantly affects the spatial distribution of CS, with the multi-year average *q* value reaching 0.356. PD exerts a strong influence, with the *q* value averaging 0.202 over the long term. The multi-year average *q* values for soil type (ST) and potential evapotranspiration (PET) are each 0.120. ST consistently exerted a medium influence annually, whereas PET’s influence diminished from medium to weak throughout the study period. The remaining five factors exert a minimal influence on the spatiotemporal heterogeneity of CS.

The driving factors of CS were interactively detected, and the results are demonstrated in Figure 10B. Between 2010 and 2022, all driving factors exhibited bivariate or nonlinear enhancement, suggesting that the interaction of any two factors provided greater explanatory power regarding the spatial heterogeneity of CS than any individual factor alone. In both 2010 and 2019, altitude (EL)∩VC was the primary explanatory factor. In 2013, PET∩VC, EL∩VC, and VC∩ST were significant explanatory factors. In 2016, PET and VC, as well as VC and ST, significantly influenced outcomes. In 2022, the interaction between PET and VC was the most significant. The interaction factors with the greatest explanatory power have varied slightly over the years; however, the combination of VC with PET, EL, and ST significantly influences CS distribution. The interactions with relatively strong explanatory power comprise AAT∩VC, AP∩VC, and VC∩PD.

## Discussion

### Reliability of CD estimation

**Figure 11:**
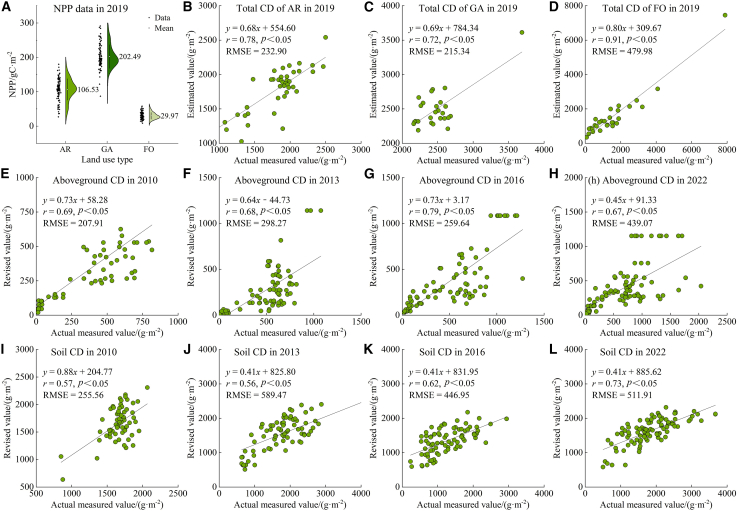
CD verification (A) NPP for 2019. (B–D) Comparison of estimated CD values and actual measured values under different LU types. (E–L) Comparison of revised CD values and actual estimated values. AR, arable land; GA, garden land; FO, forest-grassland.

CD is a critical parameter in the InVEST model, and its accuracy directly influences the precision of regional CS estimates. The CD estimation results were compared with the net primary production (NPP) data to verify the accuracy of the estimation results using the measured data. One hundred sample points were randomly selected from arable land, garden land, and forest-grassland. As shown in Figure 11A, the average NPP value was highest in garden land, followed by arable land and then forest-grassland—a pattern consistent with the aboveground CD estimates for 2019. To further assess estimation accuracy, predicted CD values for arable land, garden land, and forest-grassland were compared with measured values (Figures 11B–11D). The resulting RMSE values were 232.90, 215.34, and 479.98 gm^−2^, respectively. These errors are within an acceptable range relative to similar studies in arid regions, indicating that the estimation model performs reliably across different LU types. The relatively higher error for forest-grassland may be due to its heterogeneous structure and diverse species composition, which complicate accurate remote sensing inversion. Overall, the integration of field data with remote sensing imagery supports the rationality and accuracy of the CD estimates.

In CS assessments of similar regions, climate variables are often used to adjust CD parameters.^40^^,^^41^^,^^42^ Based on precise 2019 CD estimates, this study used temperature and precipitation to adjust CD and generate multi-temporal datasets. Additionally, AGB and SOC data collected in 2010, 2013, 2016, and 2022 were used to calculate aboveground and soil CD. The accuracy of the adjusted CD values was then verified (Figures 11E−11L). Results showed a significant positive correlation between measured and adjusted CD (*p* < 0.05), confirming the effectiveness of using field data for CD calibration.

During the experiment, to avoid damaging the ecological environment and agricultural production through root collection, below ground biomass was estimated using measured AGB combined with root-to-shoot ratios. Although these ratios are widely used in carbon assessments for similar regions, local variations may still introduce uncertainty. Furthermore, due to the difficulty of collecting full soil profile data, CD estimation was limited to the top 20 cm of soil. Future studies should include more comprehensive plant and soil sampling to improve CD accuracy. In addition, because water bodies occupy a relatively small area in the region, their carbon content was not considered in this study. Future research should address this gap by incorporating CS assessments of aquatic ecosystems.

### Response of CS to LU change

Previous studies have shown that CS is closely related to vegetation composition and LU.^34^ This study similarly found that garden land has the strongest carbon fixation capacity, followed by forest-grassland, while arable land has a relatively weaker capacity. In contrast, construction land, unused land, and water bodies exhibited significantly lower carbon fixation than the aforementioned types. Total CS declined from 2010 to 2013 but increased from 2013 to 2022, showing an overall upward trend. This pattern is primarily attributed to the reduction of unused land and the expansion of garden land and arable land. In addition, forest-grassland, as the main site of photosynthesis in the oasis-desert ecosystem, features high CD and strong carbon fixation capacity, making it a vital carbon sink in the region. The study also found that converting land types with low carbon fixation capacity into those with high carbon fixation capacity can effectively promote carbon accumulation in the oasis ecosystem, thereby increasing vegetation CS. These findings are consistent with those of Li et al.^43^ and Peng et al.^44^ regarding the relationship between LU and CS, reinforcing the conclusion that optimizing LU patterns is a key strategy for enhancing carbon sequestration in arid regions.

Scenario predictions indicate that compared to 2022, CS under the NDS, EDS, and CDS is expected to decline by 2028, while the EPS shows a projected increase. These results align with the simulated CS outcomes reported by Shao et al.^45^ and Zhu et al.^46^ Although the EPS scenario offers ecological benefits, its success depends on the continued and potentially increased allocation of water resources for ecological purposes. In water-scarce areas like the Wei-Ku Oasis, carbon sequestration goals must be carefully balanced with water resource security. Future management strategies should integrate water-use efficiency into carbon planning. Priority should be given to planting drought-tolerant species with high carbon fixation potential, strictly limiting the expansion of water-intensive crops in marginal salinization-prone areas, and optimizing irrigation methods to maximize carbon gains per unit of water used. Based on scenario simulations, this study proposes the following measurable LU planning targets (1) Maintain forest-grassland and garden land at a minimum of 35% of the total oasis area to ensure stable carbon sinks. (2) Limit the annual growth rate of construction land to less than 1.5% to minimize carbon loss. (3) When converting arable land to garden land, apply a carbon gain efficiency threshold—allowing only conversions that result in a CD increase of at least 500 gm^−2^ to ensure significant net carbon benefits. (4) Combine the expansion of arable land and garden land with water-saving irrigation measures, achieving >80% drip irrigation coverage to avoid exacerbating water scarcity. Additionally, reserve at least 15% of agricultural water for ecological purposes to support forest-grassland restoration. These quantitative targets translate research findings into actionable planning guidelines.

### Driving factors of spatiotemporal heterogeneity of CS

Using the OPGD model, this study systematically explored the driving mechanisms behind the spatiotemporal heterogeneity of CS. Factor detection analysis revealed that VC significantly influences this differentiation—a finding consistent with Liu et al.^47^ and Huang et al.^48^ Wei et al. noted substantial differences among vegetation types in VC, litter yield, and carbon fixation capacity.^49^ Vegetation growth, restoration, and expansion facilitate biomass accumulation and enhance SOC storage through litter input.^50^ An interesting finding is the decline in VC’s explanatory power from 2010 to 2022, despite an overall increase in CS. This suggests a temporal shift in the primary drivers of carbon accumulation. In the earlier period, CS dynamics were more closely linked to changes in natural and semi-natural VC. In later years, however, the increasing contribution to CS came from intensively managed human LU. Carbon dynamics in these systems are influenced by complex management practices—such as irrigation, fertilization, and crop selection—which are not fully captured by remote sensing-derived VC. As a result, VC’s relative explanatory power diminished over time. This evolution highlights the growing anthropogenic imprint on the oasis carbon cycle. In the Wei-Ku Oasis, urban expansion driven by population growth has led to a reduction in forest-grassland area, subsequently decreasing VC and CS. Meanwhile, the expansion of arable land and garden land has contributed to increased VC and regional carbon sinks, indicating that human activities exert both positive and negative feedback on CS changes. Among the various driving factors, VC serves as the core carrier of carbon input. AAT, AP, and PET represent water and heat constraints on biological growth. EL, slope (SL), and ST constitute environmental constraints. PD and gross domestic product density (GDP) reflect the intensity of human activities. CS is a comprehensive manifestation of the carbon balance within the ecosystem, collectively regulated by interactions among the atmosphere, hydrosphere, pedosphere, and biosphere.

Interaction detection revealed that two-factor interactions significantly enhance the explanatory power of individual drivers for CS heterogeneity. Specifically, the combination of VC with PET, EL, and ST strongly influences CS spatial distribution. As a key water and heat regulatory factor in arid regions, PET impacts CS by driving the water-nutrient cycle and maintaining suitable conditions for plant growth. EL and ST, as core environmental elements affecting vegetation distribution, play pivotal roles in the ecosystem’s carbon fixation process. Therefore, in advancing the “dual carbon” goals, it is essential to evaluate the combined effects of multiple driving factors on CS. Given the region’s arid conditions, water scarcity, and poor soil quality, it is recommended to prioritize drought-resistant vegetation such as *Hippophae rhamnoides Linn.*, which features high light and water use efficiency. Enhancing VC remains a viable pathway to strengthen the ecosystem’s carbon fixation capacity.

### Main findings

This study successfully achieved three core research objectives. (1) The spatiotemporal evolution characteristics of CS in the Wei-Ku Oasis were accurately depicted. The results showed that total CS exhibited a trend of “first decreasing and then increasing” from 2010 to 2022, with a net increase of 1.301 × 10^6^ t. Spatially, CS exhibited pronounced spatial gradients, decreasing from the oasis core to the surrounding desert areas. (2) Future changes in CS under four different development pathways were simulated and evaluated. By 2028, it is projected that only the EPS would lead to an increase in CS of 0.041 × 10^6^ t. In contrast, the NDS, EDS, and CDS would result in reductions of 0.547 × 10^6^, 0.159 × 10^6^, and 0.237 × 10^6^ t, respectively. Spatially, under the NDS and EDS, the expansion of construction land leads to considerable CS losses within the oasis. Under the CDS and EDS, the conversion of forest-grassland contributes to CS reductions in downstream areas and near the Tarim River. The EPS effectively maintains CS by protecting the forest-grassland. This comparison underscores the importance of actively pursuing ecological planning. (3) The main drivers and their interactions controlling the spatial heterogeneity of CS were elucidated. VC ($\bar{q}$ = 0.356) was identified as the dominant factor, followed by PD ($\bar{q}$ = 0.202). Notably, the interaction between VC and other factors ($\bar{q}$ = 0.461) was substantially stronger than that of any individual factor, with an increase in explanatory power of approximately 29.5%. This analysis reveals the complex driving mechanisms underlying CS distribution.

Multi-scenario projections offer important, policy-relevant insights. The quantitative differences highlight the substantial carbon costs associated with the NDS, EDS, and CDS, primarily driven by the encroachment of construction land and the conversion of natural and semi-natural vegetation. These development-centered scenarios are projected to cause significant carbon losses in core oasis areas and downstream regions. In contrast, the EPS demonstrates that strategic ecological conservation—especially protecting and potentially expanding forest-grassland—can reverse this trend and enhance regional CS. This contrast underscores a core socio-ecological implication: achieving carbon sequestration goals in arid oases requires actively balancing short-term economic expansion against long-term ecological security. The EPS offers a viable pathway for sustainable oasis management, aligned with the “dual carbon” goals.

### Research limitations and significance

The estimations and simulations in this study were constrained by the accuracy of input data and the assumptions inherent in the InVEST, OPGD, and machine learning models. Specifically, uncertainties arise from the use of literature-based root-to-shoot ratios, the exclusion of soil carbon below 20 cm depth, and the simplified representation of future socio-economic pathways in scenario simulations. Future research should incorporate comprehensive field measurements of root biomass and deep soil carbon profiles, integrate higher-resolution climate and LU data, and employ multi-model intercomparison frameworks to enhance the robustness and generalizability of CS assessments in arid oasis ecosystems. Moreover, exploring the coupling between water resource management and carbon sequestration will be critical for developing adaptive strategies in water-limited regions.

This study confirms the effectiveness of ecological protection measures in enhancing regional carbon sink capacity, providing a scientific basis for formulating LU policies and ecological restoration strategies. The findings suggest that optimizing the spatial structure of LU to increase VC is an effective strategy for enhancing the carbon sink potential of arid oases and supporting climate change mitigation. Furthermore, the quantitative comparisons among different development scenarios highlight the tangible carbon costs associated with conventional expansion-oriented pathways, offering actionable insights for balancing socioeconomic development with ecological security in water-limited arid regions.

## Resource availability

### Lead contact

Further information and requests for resources and data should be directed to and will be fulfilled by the lead contact, Xuemei Wang (xmwang2022@xjnu.edu.cn).

### Materials availability

This study did not generate new unique reagents.

### Data and code availability

• All the data reported in this paper will be shared by the lead contact upon request.
• This paper does not report original code.
• Any additional information required to reanalyze the data reported in this paper is available from the lead contact upon request.

## Acknowledgments

This study was supported by the 10.13039/501100001809National Natural Science Foundation of China (42461042 and 41561051) and the 10.13039/100009110Natural Science Foundation of Xinjiang Uygur Autonomous Region, China (2023D01A44).

## Author contributions

Conceptualization, software, methodology, writing – original draft, B.A.; investigation, data curation, supervision, writing – review and editing, and funding acquisition, X.W.; supervision and writing – review and editing, C.L.; data curation, X.F. All authors have read and agreed to the published version of the manuscript.

## Declaration of interests

The authors declare no competing interests.

## STAR★Methods

### Key resources table

**Table:** 

REAGENT or RESOURCE	SOURCE	IDENTIFIER
**Deposited data**		

LU data	Scientific reports	https://www.nature.com/articles/s41598-024-79539-0
Sentinel 2	Google earth engine	https://earthengine.google.com/
MOD15A2H	Google earth engine	https://earthengine.google.com/
MOD16A2GF	Google earth engine	https://earthengine.google.com/
MOD17A3HGF	Google earth engine	https://earthengine.google.com/
SRTM V3	Google earth engine	https://earthengine.google.com/
China regional vegetation cover dataset	National Tibetan Plateau Data Center	https://data.tpdc.ac.cn/
Monthly mean temperature dataset for China	National Tibetan Plateau Data Center	https://data.tpdc.ac.cn/
Monthly precipitation dataset for China	National Tibetan Plateau Data Center	https://data.tpdc.ac.cn/
CIMP6 China future climate scenario annual dataset	Resource and Environmental Science Data Platform	https://www.resdc.cn/
Spatial distribution data of soil types in China	Resource and Environmental Science Data Platform	https://www.resdc.cn/
LandScan population data	Oak Ridge National Laboratory	https://landscan.ornl.gov/
“DMSP-OLS-like” nighttime light dataset	Harvard Dataverse	https://dataverse.harvard.edu/
Real gross domestic product dataset	Figshare	https://figshare.com/

**Software and algorithms**		

ArcGIS 10.8	ESRI	https://www.arcgis.com/index.html
Origin 2021	OriginLab	https://www.originlab.com/
InVEST model	Natural Capital Project	https://naturalcapitalproject.stanford.edu/software/invest
OPGD model	Geodetector	http://www.geodetector.cn/

### Method details

#### Study area

The Wei-Ku Oasis is located on the northern edge of the Tarim Basin in Xinjiang, China, and is an important oasis in the arid northwestern region of China. Its extent is defined by the coordinates spanning from 40°57′12″N to 41°47′48″N and from 82°04′59″E to 83°42′20″E. Administratively, it falls under the jurisdiction of Aksu Prefecture, Xinjiang Uygur Autonomous Region, covering mainly the oasis areas of Kuqa City, Shaya County, and Xinhe County. The landform is a typical fan-shaped plain oasis. The terrain is relatively flat and open, experiencing a gradual decline in altitude from the northwesterly to the southeasterly direction. Due to its location in the inland hinterland of China, this area falls within a temperate continental arid climate zone, exhibiting cold winters, hot summers, scarce precipitation, vigorous evaporation, and frequent sandstorms. The Weigan River, the Kuqa River, and the Tarim River are the three major agricultural irrigation water sources of this oasis. In recent years, affected by population growth and accelerated urbanization, the LU structure of the oasis has undergone significant changes, which in turn have had a remarkable impact on its ecosystem CS. Therefore, scientifically evaluating the current status of CS in the oasis ecosystem, systematically analyzing the driving mechanism of CS spatial differentiation, and reasonably predicting the future development trend of CS hold substantial practical value for strengthening the carbon sink potential of oasis ecosystems and promoting the coordinated development of human-land relations in arid areas.

#### Data sources

##### Actual measurement data

The research team conducted vegetation and soil surveys in the study area in July of 2010, 2013, 2016, 2019, and 2022. During the survey process, detailed site information of the sample plots was recorded, including longitude and latitude coordinates, vegetation types, and soil texture. The sample sizes for the five periods were 60, 78, 80, 96, and 95, respectively. At each sample point, a 50 m × 50 m large vegetation plot was surveyed; these plots were mainly composed of artificial vegetation and natural vegetation. For each artificial vegetation sample plot (i.e., arable land or garden land), three 10 m × 10 m small crop subplots were surveyed. For natural vegetation sample plots (i.e., forest-grassland), three 10 m × 10 m subplots were surveyed targeting tree, shrub, and grassland communities, respectively, along with five 1 m × 1 m herbaceous subplots. During the survey, standard plants of each species were selected, and their plant height, crown width, and plant count were recorded. Standard branches of uniform size were cut from each standard plant, and their branches and leaves were separated. The fresh weight was measured using a balance with an accuracy of 0.01 g. Subsequently, the samples were placed in kraft paper bags and sent to the laboratory, where they were dried in a constant-temperature drying oven set at 80 °C. After cooling, the samples were weighed, and the moisture content was determined using the dry and fresh weights of the standard branches. Based on this, the aboveground biomass (AGB) of the standard plants was calculated, thereby allowing the estimation of AGB for each sample plot based on the abundance of each species.

Meanwhile, a soil survey was also conducted using the plum-blossom sampling method. Five soil samples were collected from each 10 m × 10 m subplot at a depth of 0–20 cm. After the five soil samples were thoroughly mixed, approximately 300 g of the mixture was placed in numbered self-sealing bags and sealed. A total of 60, 78, 80, 96, and 95 soil samples were collected for the five survey periods, respectively. After being transported back to the laboratory, the soil samples were thoroughly air-dried at room temperature, ground, and passed through a 100-mesh sieve. The soil organic carbon (SOC) content was determined using the potassium dichromate oxidation method. The measured AGB and SOC values for the study area across the multiple survey periods are presented in the below table.

**Table:** Measured values of AGB and SOC

Survey year	AGB/(g·m^−2^)			SOC/(g·kg^−1^)		
	Range	Median	Average	Range	Median	Average
2010	12.92–816.25	457.89	396.11	2.78–6.76	5.52	5.44
2013	5.90–1074.35	565.58	481.34	2.16–13.96	6.05	6.38
2016	19.69–1273.54	578.53	521.34	0.76–9.62	3.89	4.11
2019	5.83–3416.52	331.99	437.74	0.67–10.20	4.91	4.99
2022	11.14–2041.14	678.75	625.79	1.64–30.34	6.12	6.95

##### Remote sensing data

This study utilized diverse remote sensing data (see table below) to produce the necessary information for CS assessment and analysis of driving forces. All data were standardized to the WGS_1984_UTM_Zone_44N coordinate system, and the spatial resolution resampling was uniformly set to 10m.

**Table:** Remote sensing data

Data	Spatial resolution	Data source
LU data	10 m	An et al.^51^
Sentinel 2	10 m/20 m	https://earthengine.google.com/
MOD15A2H	500 m	–
MOD16A2GF	500 m	–
MOD17A3HGF	500 m	–
SRTM V3	30 m	–
China regional vegetation cover dataset	250 m	https://data.tpdc.ac.cn/
Monthly mean temperature dataset for China	1 km	–
Monthly precipitation dataset for China	1 km	–
CIMP6 China future climate scenario annual dataset	50 km	https://www.resdc.cn/
Spatial distribution data of soil types in China	1 km	–
LandScan population data	1 km	https://landscan.ornl.gov/
“DMSP-OLS-like” nighttime light dataset	1 km	https://dataverse.harvard.edu/
Real gross domestic product dataset	1 km	https://figshare.com/

The LU data for the Wei-Ku Oasis utilized in this study were classified independently by An et al.^51^ using field survey data and remote sensing images. The multi-phase data from 2010 to 2022 exhibit an overall accuracy exceeding 90% and a Kappa coefficient of 0.88. The 2028 LU forecast data encompass the natural development (NDS), economic development (EDS), cropland development (CDS), and ecological protection (EPS) scenarios. In NDS, LU change continues the historical trend. Both EDS and CDS focus on development, but EDS focuses mainly on the expansion of construction land, while CDS focuses on the expansion of arable land. EPS focuses on forest-grassland ecological protection. The CD data is predominantly derived from our initial estimates of AGB and SOC. A total of 44 variables were utilized for estimating AGB and SOC (see table below). These variables include: the original bands, soil indices, vegetation indices, image enhancement features, and texture features from Sentinel-2; data products such as leaf area index (LAI), fraction of photosynthetically active radiation (FPAR), evapotranspiration (ET), potential evapotranspiration (PET), latent heat flux (LE), and potential latent heat flux (PLE) from the MOD15A2H and MOD16A2GF datasets; environmental factors, including altitude (EL), slope (SL), and aspect (AS), extracted from SRTM V3 data; and the annual average temperature (AAT) and annual precipitation (AP), calculated based on monthly average temperature and precipitation datasets. The temperature and precipitation data, both historical and projected, utilized for CD correction were derived from the processing of monthly mean temperature datasets, monthly precipitation datasets, and the CIMP6 future climate scenario annual dataset. The investigation into the key determinants of the geographical variation in CS identified factors from two domains: seven natural factors, namely AAT, AP, PET, EL, SL, vegetation cover (VC), and soil type (ST), and two human factors, namely population density (PD) and gross domestic product density (GDP).

**Table:** Variables used for the estimation of AGB and SOC

Variable Types	Variables for AGB estimation	Variables for SOC estimation	Reference
Original bands	B2, B3, B4, B5, B6, B7, B8, B8A, B11, B12		Chan et al.^52^; Sunantha et al.^53^
Soil index	/	BI, BI2, CI, RI, SI, NDSI	Deng et al.^54^; Li et al.^55^
Vegetation index	NDVI, kNDVI, RVI, DVI, SAVI, MSAVI, ARVI, S2REP, REIP		Guo et al.^56^; Li et al.^57^
Vegetation products	LAI, FPAR, ET, PET, LE, PLE	/	Song et al.^58^; Kerebeh et al.^59^
Image enhancement	ALL, VIS, RED, PCA1, PCA2, PCA3		Liu et al.^60^; Wu et al.^61^
Texture features	ASM, CON, COR, DIS, ENT, IDM, SAV, VAR		Hernández-Martínez et al.^62^; Liu et al.^63^
Environmental factors	EL, SL, AS, AAT, AP		Ho et al.^64^; Hu B et al.^65^

ALL = B2+B3+B4+B5+B6+B7+B8+B8A + B11 + B12; VIS = B2+B3+B4; RED = B5+B6+B7+B8A; PCA1, PCA2, and PCA3 are the top three principal components of principal component analysis, respectively.

#### Methods for estimating AGB and SOC

##### Sample division and variable selection

The Sample Set Partitioning based on Joint X-Y Distance (SPXY) algorithm is a method for sample partitioning derived from the Kennard-Stone algorithm. The analysis also examines the link between the explanatory and outcome indicators, which can enhance the differences and representativeness among samples, thus improving the model’s stability.^66^ The Pearson correlation coefficient (PCC), the Competitive Adaptive Reweighted Sampling (CARS), and the Iterative Retained Information Variable (IRIV) algorithm were selected for variable screening. First, PCC was used for preliminary correlation screening, which could quickly eliminate a large number of obviously irrelevant variables and reduce the complexity of subsequent calculations. Then, CARS and IRIV were used for secondary fine screening, which may obtain a variable set with better performance than the simple filtering method (PCC), or may mistakenly eliminate some useful weak information variables during operation. The specific effect of secondary screening needs to be analyzed in combination with modeling accuracy. In short, PCC is a coarse screen in the modeling stage, which greatly reduces the data dimension before modeling, while CARS and IRIV are more refined choices in the modeling stage. Their combination can effectively overcome the limitations of a single method in terms of collinearity and high-dimensional calculation, and mine the most valuable streamlined variable set, thereby promoting the construction of a high-performance estimation model. The PCC quantifies the strength and direction of the correlation between two indicators.^67^ This correlation is usually represented by the correlation coefficient (*r*), with a value range of −1 to 1. If −1≤*r* < 0, the two indicators exhibit a negative correlation; if *r* = 0, the indicators are uncorrelated; and if 0 < *r* ≤ 1, the indicators demonstrate a positive correlation. The CARS algorithm emulates the “survival of the fittest” principle from Darwin’s theory for variable selection.^68^ The approach integrates the Monte Carlo sampling method with the partial least-squares regression model to determine variable weights, employing adaptive reweighted sampling technology and an exponential decay function to remove variables with low weights. The model is constructed using the new set of variables, and the calculations are reiterated to identify the optimal variable combination that minimizes the root-mean-square error during cross-validation. The IRIV algorithm employs a binary matrix shuffling filter, utilizing random variable combinations while thoroughly accounting for variable correlations during selection.^69^ In various operations, non-information and interference variables are removed, while strong and weak variables are preserved. Ultimately, reverse elimination is conducted to identify the optimal variable combination.

##### Regression model and estimation accuracy evaluation

Random forest regression (RFR) employs Bootstrap resampling techniques to generate samples for the training set and constructs multiple decision trees to create a random forest.^70^ In RFR, each decision tree is trained and predicted independently, which can effectively reduce the risk of overfitting. RFR averages or computes weighted averages of the prediction outcomes from multiple decision trees to derive the final regression result. Support vector machine regression (SVMR) is a regression algorithm derived from support vector machine principles. The kernel function in SVMR is crucial as it transforms the input vector from the starting domain to a high-dimensional domain for computation. SVMR effectively addresses nonlinear regression issues and is extensively applied across multiple domains.^71^

The coefficient of determination (*R*^2^), root-mean-square error (RMSE), relative percent deviation (RPD), and 1:1 line are employed to evaluate the model’s estimation capability. A higher *R*^2^, approaching 1, correlates with a lower RMSE and a greater RPD, indicating improved model estimation accuracy. When RPD is less than 1.4, the model exhibits poor estimation performance on the sample; when RPD is between 1.4 and 2, the model provides a rough estimation of the sample; when RPD is 2 or higher, the model demonstrates strong estimation capability for the sample. The 1:1 line evaluates the extent of deviation between the points formed by the measured data and the estimated data relative to the line y = x. Proximity of the sample point to the 1:1 line correlates positively with estimation accuracy. The formulas for calculating *R*^2^, RMSE, and RPD are presented below:

(Equation 1) $$R^{2}=1-\frac{\sum_{i=1}^{n}{\left(z_{i}-{\hat{z}}_{i}\right)}^{2}}{\sum_{i=1}^{n}{\left(z_{i}-\bar{z}\right)}^{2}}$$

(Equation 2) $$RMSE=\sqrt{\frac{1}{n}\sum_{i=1}^{n}{\left(z_{i}-{\hat{z}}_{i}\right)}^{2}}$$

(Equation 3) $$SD=\sqrt{\frac{1}{n-1}\sum_{i=1}^{n}{\left(z_{i}-\bar{z}\right)}^{2}}$$

(Equation 4) $$RPD=\frac{SD}{RMSE}$$

where *n* is the quantity of specimens; *z*_*i*_ is the observed value of the specimen; ${\hat{z}}_{i}$ is the estimated value of the specimen; $\bar{z}$ is the mean of the observed values of the specimen; and *SD* is the standard deviation.

#### CD estimation method

##### CD calculation

Estimation of aboveground and underground CD in arable land, garden land, forest-grassland, and unused land: The aboveground CD was determined by converting the AGB inversion results using a carbon conversion coefficient of 0.45. The underground CD was estimated by combining the root-shoot ratio, the carbon conversion coefficient (0.45), and the AGB inversion results. The determination of the root-shoot ratio was based on the research findings of various scholars in arid and semi-arid regions (see table below). Based on the reasonable range proposed by relevant scholars, the root-shoot ratios of arable land, garden land, forest-grassland, and unused land in the study area were determined to be 0.10, 0.30, 3.70, and 4.50, respectively.

**Table:** Reference values of the root-to-shoot ratio

Type	Root-to-shoot ratio	Reference
Arable land	0.10	Huang et al.^72^; Zhang et al.^73^
Garden land	0.21	Chen et al.^74^
	0.17–0.36	Huang et al.^72^
Forest-grassland	1.24–2.42	Wang et al.^75^
	3.61	Zhang et al.^76^
	3.70	Jackson et al.^77^
	2.00–14.30	Yang et al.^78^; Ma et al.^79^
	8.70 (±5.16)	Fan et al.^80^
Unused land	4.50	Huang et al.^72^
	3.04 (±4.41)	Fan et al.^80^

The soil CD of arable land, garden land, forest-grassland, and unused land was estimated through field sampling and calculation. A total of 70 soil bulk density data points were collected in the field. To improve calculation efficiency, these data were categorized by LU type and averaged. The final high-precision soil bulk densities obtained were 1.53 g cm^−3^ for arable land, 1.48 g cm^−3^ for garden land, 1.71 g cm^−3^ for forest-grassland, and 1.24 g cm^−3^ for unused land. Soil CD was estimated using formula 5, based on the SOC inversion results, soil layer thickness, and soil bulk density.

(Equation 5) SCD=S×B×G×0.01

where *SCD* is the soil CD (kg·m^−2^); *S* is the SOC content (g·kg^−1^); *B* is the soil bulk density (g·cm^−3^); and *G* is the soil layer thickness (cm).

Estimation of CD of construction land: Considering that construction land is mainly buildings and roads, while parks and green spaces have forest-grassland. This estimation utilized the green coverage rate as a reference point. In 2019, the green coverage rate of construction land in the Aksu region was 41.4%. The CD of construction land was calculated by multiplying the CD of forest-grassland with the green coverage rate.

Estimation of dead organic CD for each LU type: Delaney et al.^81^ determined that the dead organic CD is approximately one-tenth that of aboveground CD. Consequently, this study adopts this ratio as a standard for estimating dead organic CD.

##### CD correction

Domestic and foreign studies^82^^,^^83^^,^^84^ have shown that both biomass CD and soil CD are significantly positively correlated with precipitation. Therefore, the linear regression model (Formula (6)) and exponential regression model (Formula (7)) in the study of Alam et al.^82^ are used to correct soil CD and biomass CD. The correction formula for the temperature factor on biomass CD is the linear regression model (Formula (8)) in the study of Giardina and Ryan^83^ and Chen et al.^84^ Nevertheless, the association between temperature and soil CD is substantially less pronounced than that between precipitation and soil CD, and there is currently no detailed literature on the relationship between temperature and soil CD, so the effect of temperature on soil CD is not considered. Temperature and precipitation use historical and predicted data. This study matches future data from SSP126, SSP245, SSP370, and SSP585 within the CIMP6 climate scenario with EPS, NDS, CDS, and EDS projections for the year 2028. SSP126 represents a low emission concentration and sustainable development trajectory, reflecting a heightened awareness of environmental protection, akin to EPS. SSP245 exhibits a medium emission concentration, with socioeconomic factors aligning with its historical trend, akin to NDS. The carbon emissions associated with SSP370 have shown a consistent upward trend, with an increasing emphasis on the national food supply, analogous to CDS. SSP585 illustrates elevated carbon emissions alongside swift economic growth, aligning with EDS. The formula for correction is outlined as follows:

(Equation 6) $$D_{SP}=3.3968\times P+3996.1$$

(Equation 7) $$D_{BP}=6.798\times e^{0.0054\times P}$$

(Equation 8) $$D_{BT}=28\times T+398$$

(Equation 9) $$C_{S}=\frac{D_{SP}^{\prime }}{D_{SP}^{\prime \prime }}$$

(Equation 10) $$C_{BP}=\frac{D_{BP}^{\prime }}{D_{BP}^{\prime \prime }}$$

(Equation 11) $$C_{BT}=\frac{D_{BT}^{\prime }}{D_{BT}^{\prime \prime }}$$

(Equation 12) $$C_{B}=C_{BP}\times C_{BT}$$

where *P* is precipitation; *T* is temperature; *D*_*SP*_ is soil CD corrected by precipitation; *D*_*BP*_ and *D*_*BT*_ are biomass CD corrected by precipitation and temperature, respectively; *C*_*S*_ and *C*_*B*_ are correction coefficients of soil CD and biomass CD, respectively; *C*_*BP*_ and *C*_*BT*_ are precipitation and temperature correction coefficients of biomass CD, respectively; $D_{SP}^{\prime }$ and $D_{SP}^{\prime \prime }$ are soil CD corrected by precipitation for the current and reference times, respectively; $D_{BP}^{\prime }$ and $D_{BP}^{\prime \prime }$ are biomass CD corrected by precipitation for the current and reference times, respectively; $D_{BT}^{\prime }$ and $D_{BT}^{\prime \prime }$ are biomass CD corrected by temperature for the current and reference times, respectively.

#### InVEST model

The Carbon Storage and Sequestration module within the InVEST model facilitates the estimation of CS. This module primarily requires data on LU and CD.^85^ The model’s formula is presented as follows:

(Equation 13) $$D_{i}=D_{i,a}+D_{i,b}+D_{i,s}+D_{i,d}$$

(Equation 14) $$S_{total}=\sum_{i=1}^{n}D_{i}\times A_{i}$$

where *i* is a certain LU type; *D*_*i*_ is the total CD of *i*; *D*_*i*,*a*_ is the aboveground CD of *i*; *D*_*i*,*b*_ is the underground CD of *i*; *D*_*i*,*s*_ is the soil CD of *i*; *D*_*i*,*d*_ is the dead organic CD of *i*; *S*_*total*_ is the total CS; *A*_*i*_ is the total area of *i*; *n* is the total number of LU types.

#### OPGD model

OPGD serves as a tool to assess the underlying factors leading to the spatial heterogeneity of geographic variables.^86^ Factor detection is fundamental, and the *q* value (formula 15) serves to assess the role of factors in driving the spatial diversity of CS. An elevated *q* value suggests enhanced interpretive strength. Interaction detection assesses the explanatory capacity of the joint influence of two factors on CS.^87^

(Equation 15) $$q=1-\frac{\sum_{i=1}^{N}A_{i}\sigma_{i}^{2}}{A\sigma^{2}}\;q\in \left[0,1\right]$$

where *q* is the explanatory power of the independent variable; *i* = 1,⋯,*N* is the zoning of LU types or influencing factors; *A*_*i*_ and *A* are the quantity of components in sub-district *i* and the entire region, respectively; $\sigma_{i}^{2}$ and *σ*^2^ are the variances of LU types in sub-district *i* and the entire region, respectively.

### Quantification and statistical analysis

All statistical analyses were performed in ArcGIS 10.8 and Origin 2021. The specific tests used are clearly listed in the figures and tables.
